# Supplementary material for: Functional Characterization of Three GnRH Isoforms in Small Yellow Croaker Larimichthys polyactis Maintained in Captivity: Special Emphasis on Reproductive Dysfunction
Source: Biology (Basel). 2022 Aug 10;11(8):1200. doi: 10.3390/biology11081200 (PMC9404844; doi:10.3390/biology11081200)
Supplement: Supplementary file 1 [file biology-11-01200-s001.zip › Supplementary Table S1.pdf]

Supplementary Table S1. List of primers used for full length cDNA cloning of three isoform of GnRH gene in small yellow croaker.

Primer Name	Nucleotide sequence (5′--- 3′)	Purpose
GnRH-1–Fw	AAGCATGGCTCCTCAGACG	RT-PCR (Partial Sequencing)
GnRH-1–Rv	GTCCAGAAATCCTTTCATCCT	
GnRH-2–Fw	TTGGGCTGCTTCTATGTGC	
GnRH-2–Rv	GCTTTCACTTCCTCTTCTGG	
GnRH-3–Fw	AAGTGAGCAGCAGAGTGATG	
GnRH-3–Rv	CTTTCGGTCAAAATGACTGGA	
crk-GnRH-1 –3′RACE	GATTACGCCAAGCTTGCACTGGTCGTATGGACTGAGTCCA	RACE PCR
crk-GnRH-1–5′RACE	GATTACGCCAAGCTTTCAGAACGCTGCAGGGCGAGTCCA	
crk-GnRH-2 –3′RACE	GATTACGCCAAGCTTGAGTGCCCAAGCCCAAGAGAGACTTAG	
crk-GnRH-2–5′RACE	GATTACGCCAAGCTTCAGCTGAGCTCCCACACATAGAAGTAGC	
crk-GnRH-3 –3′RACE	GATTACGCCAAGCTTGTATGGTCTAAGTCTCTCTTGGGCTTGG	
crk-GnRH-3–5′RACE	GATTACGCCAAGCTTCCAACGCCAACAACAACACCTGCACC	
Universal Primer (Short)	CTAATACGACTCACTATAGGGC	
Universal Primer (Long)	CTAATACGACTCACTATAGGGCAAGCAGTGGTATCAACGCAGAGT	
Oligo dT (OdT)	GGCCACGCGTCGACTAGTACTTTTTTTTTTTTTTTTT	cDNA Synthesis
Oligo dT Adaptor (AP)	GGCCACGCGTCGACTAGTAC	

Molecular, Evolutionary and Functional Characterization of Three GnRH isoforms in Small Yellow Croaker *Larimichthys polyactis* Maintained in Captivity
