# Supplementary material for: Functional Characterization of Three GnRH Isoforms in Small Yellow Croaker Larimichthys polyactis Maintained in Captivity: Special Emphasis on Reproductive Dysfunction
Source: Biology (Basel). 2022 Aug 10;11(8):1200. doi: 10.3390/biology11081200 (PMC9404844; doi:10.3390/biology11081200)
Supplement: Supplementary file 1 [file biology-11-01200-s001.zip › Supplementary Table S2.pdf]

Supplementary Table S2. Sequence information of GnRHs used for motif analysis of three GnRH isoforms.

GnRH Isoform	Common Name	Scientific Name	GenBank Accession No.	
			Nucleotide	Protein
GnRH1	Small yellow croaker	*Larimichthys polyactis*	OK042349.1	UFT26658.1
	Large yellow croaker	*Larimichthys crocea*	NM_001303356.1	NP_001290285.1
	Atlantic croaker	*Micropogonias undulatus*	AY324668.2	AAQ16501.2
	Red seabream	*Pagrus major*	D86582.1	BAA13129.1
	Chub mackerel	*Scomber japonicus*	HQ108193.1	ADP89591.1
	African cichlid	*Haplochromis burtoni*	AF076961.4	AAC27716.1
	Tilapia	*Oreochromis niloticus*	AB101665.1	BAC56849.1
GnRH2	Small yellow croaker	*Larimichthys polyactis*	OK042350.1	UFT26659.1
	Large yellow croaker	*Larimichthys crocea*	KM232949.1	AIU45792.1
	Atlantic croaker	*Micropogonias undulatus*	AY324669.2	AAQ16502.2
	Chub mackerel	*Scomber japonicus*	HQ108194.1	ADP89592.1
	African cichlid	*Haplochromis burtoni*	AF076962.1	AAC27717.1
	Tilapia	*Oreochromis niloticus*	AB101666.1	BAC56850.1
GnRH3	Small yellow croaker	*Larimichthys polyactis*	OK042351.1	UFT26660.1
	Large yellow croaker	*Larimichthys crocea*	KP159744.1	AJY54663.1
	Atlantic croaker	*Micropogonias undulatus*	AY324670.2	AAQ16503.2
	Red seabream	*Pagrus major*	D26108.1	BAA05104.1
	Chub mackerel	*Scomber japonicus*	HQ108195.1	ADP89593.1
	African cichlid	*Haplochromis burtoni*	AF076963.1	AAC27718.1
	Tilapia	*Oreochromis niloticus*	AB104863.2	BAC65156.1
