# Supplementary material for: Functional Characterization of Three GnRH Isoforms in Small Yellow Croaker Larimichthys polyactis Maintained in Captivity: Special Emphasis on Reproductive Dysfunction
Source: Biology (Basel). 2022 Aug 10;11(8):1200. doi: 10.3390/biology11081200 (PMC9404844; doi:10.3390/biology11081200)
Supplement: Supplementary file 1 [file biology-11-01200-s001.zip › Supplementary Table S3.pdf]

Supplementary Table S3. Sequence information of GnRHs used for multiple sequence alignment of three GnRH isoforms.

GnRH Isoform	Common Name	Scientific Name	GenBank Accession No.	
			Nucleotide	Protein
GnRH1	Small yellow croaker	*Larimichthys polyactis*	OK042349.1	UFT26658.1
	Atlantic croaker	*Micropogonias undulatus*	AY324668.2	AAQ16501.2
	Large yellow croaker	*Larimichthys crocea*	NM_001303356.1	NP_001290285.1
	Japanese anchovy	*Engraulis japonicus*	JX406273.1	AFO70217.1
	African catfish	*Clarias gariepinus*	X78049.1	CAA54971.1
	Japanese eel	*Anguilla japonica*	AB026989.1	BAA82608.1
	Lake whitefish	*Coregonus clupeaformis*	AY245104.2	AAP57221.1
	Japanese Medaka	*Oryzias latipes*	AB041333.1	BAB16303.1
	Red seabream	*Pagrus major*	D86582.1	BAA13129.1
GnRH2	Small yellow croaker	*Larimichthys polyactis*	OK042350.1	UFT26659.1
	Atlantic croaker	*Micropogonias undulatus*	AY324669.2	AAQ16502.2
	Large yellow croaker	*Larimichthys crocea*	KM232949.1	AIU45792.1
	Japanese anchovy	*Engraulis japonicus*	JX406274.1	AFO70218.1
	African catfish	*Clarias gariepinus*	X78047.1	CAA54969.1
	Japanese eel	*Anguilla japonica*	AB026990.1	BAA82609.1
	Lake whitefish	*Coregonus clupeaformis*	AY245102.1	AAP57219.1
	Japanese Medaka	*Oryzias latipes*	AB041330.1	BAB16300.1
GnRH3	Small yellow croaker	*Larimichthys polyactis*	OK042351.1	UFT26660.1
	Large yellow croaker	*Larimichthys crocea*	KP159744.1	AJY54663.1
	Atlantic croaker	*Micropogonias undulatus*	AY324670.2	AAQ16503.2
	Japanese anchovy	*Engraulis japonicus*	JX406275.1	AFO70219.1
	Red seabream	*Pagrus major*	D26108.1	BAA05104.1
	Lake whitefish	*Coregonus clupeaformis*	AY245103.1	AAP57220.1
	Japanese Medaka	*Oryzias latipes*	AB041332.1	BAB16302.1
