# Supplementary material for: Functional Characterization of Three GnRH Isoforms in Small Yellow Croaker Larimichthys polyactis Maintained in Captivity: Special Emphasis on Reproductive Dysfunction
Source: Biology (Basel). 2022 Aug 10;11(8):1200. doi: 10.3390/biology11081200 (PMC9404844; doi:10.3390/biology11081200)
Supplement: Supplementary file 1 [file biology-11-01200-s001.zip › Supplementary Table S4.pdf]

Supplementary Table S4. Sequence information of GnRHs used for phylogenetic analysis of three GnRH isoforms.

GnRH Isoform	Common Name	Scientific Name	GenBank Accession No.	
			Nucleotide	Protein
GnRH1	Small yellow croaker	*Larimichthys polyactis*	OK042349.1	UFT26658.1
	Large yellow croaker	*Larimichthys crocea*	NM_001303356.1	NP_001290285.1
	Atlantic croaker	*Micropogonias undulatus*	AY324668.2	AAQ16501.2
	Japanese anchovy	*Engraulis japonicus*	JX406273.1	AFO70217.1
	American shad	*Alosa sapidissima*	AF536381.1	AAN04492.1
	Japanese eel	*Anguilla japonica*	AB026989.1	BAA82608.1
	Lake whitefish	*Coregonus clupeaformis*	AY245104.2	AAP57221.1
	European seabass	*Dicentrarchus labrax*	AF224279.1	AAF62898.1
	Medaka	*Oryzias latipes*	AB041333.1	BAB16303.1
	African cichlid	*Haplochromis burtoni*	AF076961.4	AAC27716.1
	Tilapia	*Oreochromis niloticus*	AB101665.1	BAC56849.1
	Chub mackerel	*Scomber japonicus*	HQ108193.1	ADP89591.1
	Red seabream	*Pagrus major*	D86582.1	BAA13129.1
	Pejerrey	*Odontesthes bonariensis*	AY744689.1	AAU94309.2
	Swamp eel	*Monopterus albus*	AY858056.1	AAW51121.1
	African catfish	*Clarias gariepinus*	X78049.1	CAA54971.1
GnRH2	Small yellow croaker	*Larimichthys polyactis*	OK042350.1	UFT26659.1
	Large yellow croaker	*Larimichthys crocea*	KM232949.1	AIU45792.1
	Atlantic croaker	*Micropogonias undulatus*	AY324669.2	AAQ16502.2
	Japanese anchovy	*Engraulis japonicus*	JX406274.1	AFO70218.1
	Japanese eel	*Anguilla japonica*	AB026990.1	BAA82609.1
	Lake whitefish	*Coregonus clupeaformis*	AY245102.1	AAP57219.1
	Medaka	*Oryzias latipes*	AB041330.1	BAB16300.1
	African cichlid	*Haplochromis burtoni*	AF076962.1	AAC27717.1
	Tilapia	*Oreochromis niloticus*	AB101666.1	BAC56850.1
	Chub mackerel	*Scomber japonicus*	HQ108194.1	ADP89592.1
	European eel	*Anguilla anguilla*	GU332293.1	ADD92005.1
	Pejerrey	*Odontesthes bonariensis*	AY744687.1	AAU94307.1
	Swamp eel	*Monopterus albus*	AY858054.1	AAW51119.1
	African catfish	*Clarias gariepinus*	X78047.1	CAA54969.1
	Chanchita	*Cichlasoma dimerus*	HQ444961.1	ADV31310.1
	Zebrafish	*Danio rerio*	AY657018.1	AAU43784.1
GnRH3	Small yellow croaker	*Larimichthys polyactis*	OK042351.1	UFT26660.1
	Large yellow croaker	*Larimichthys crocea*	KP159744.1	AJY54663.1
	Atlantic croaker	*Micropogonias undulatus*	AY324670.2	AAQ16503.2
	Japanese anchovy	*Engraulis japonicus*	JX406275.1	AFO70219.1
	Lake whitefish	*Coregonus clupeaformis*	AY245103.1	AAP57220.1
	European seabass	*Dicentrarchus labrax*	AF224280.1	AAF62899.1
	Medaka	*Oryzias latipes*	AB041332.1	BAB16302.1
	African cichlid	*Haplochromis burtoni*	AF076963.1	AAC27718.1
	Tilapia	*Oreochromis niloticus*	AB104863.2	BAC65156.1
	Chub mackerel	*Scomber japonicus*	HQ108195.1	ADP89593.1
	Red seabream	*Pagrus major*	D26108.1	BAA05104.1
	Pejerrey	*Odontesthes bonariensis*	AY744688.1	AAU94308.1
	Swamp eel	*Monopterus albus*	AY858055.1	AAW51120.1
	Chanchita	*Cichlasoma dimerus*	HQ444962.2	ADV31311.2
	Zebrafish	*Danio rerio*	AJ304429.1	CAC18539.1
