# Supplementary material for: Functional Characterization of Three GnRH Isoforms in Small Yellow Croaker Larimichthys polyactis Maintained in Captivity: Special Emphasis on Reproductive Dysfunction
Source: Biology (Basel). 2022 Aug 10;11(8):1200. doi: 10.3390/biology11081200 (PMC9404844; doi:10.3390/biology11081200)
Supplement: Supplementary file 1 [file biology-11-01200-s001.zip › Supplementary Table S5.pdf]

Supplementary Table S5. List of primers used for qRT-PCR analysis of three GnRH isoforms and three GtH subunits in small yellow croaker.

Primer Name	Nucleotide sequence (5'--- 3')	Accession No.	Size (bp)
crk-GnRH-1 (RT) Fw	TCCTCAAACGTCGGCCATG	OK042349.1	177
crk-GnRH-1 (RT) Rv	CAGTTCAGAACGCTGCAGG		
crk-GnRH-2 (RT) Fw	ATGTGTGGGAGCTCAGCTG	OK042350.1	198
crk-GnRH-2 (RT) Rv	TCCCTGGCTAAGGCATCCA		
crk-GnRH-3 (RT) Fw	TGATGGTGCAGGTGTTGTTG	OK042351.1	186
crk-GnRH-3 (RT) Rv	CTAAGTCTCTCTTGGGCTTG		
crk-FSHβ (RT) Fw	GGCAACACCGAGTTCATCG	MT239441.1	199
crk- FSHβ (RT) Rv	CGTTGCATGCGCTACACTC		
crk-LHβ (RT) Fw	CATCACCAAGGACCCTGTC	MW192797.1	198
crk- LHβ (RT) Rv	CTCTCGAAGGTGCTGTCAG		
crk-GPα (RT) Fw	TGCACACTGAGCAAGAACAG	MW192796.1	178
crk-GPα (RT) Rv	CCTCTATCTCGTAGCTGTGC		
crk-β-Actin (RT) Fw	GCTGTCTTCCCATCCATCG	MT330378	193
crk-β-Actin (RT) Rv	CGTTGTAGAAGGTGTGATGC		
