# Supplementary material for: Functional Characterization of Three GnRH Isoforms in Small Yellow Croaker Larimichthys polyactis Maintained in Captivity: Special Emphasis on Reproductive Dysfunction
Source: Biology (Basel). 2022 Aug 10;11(8):1200. doi: 10.3390/biology11081200 (PMC9404844; doi:10.3390/biology11081200)
Supplement: Supplementary file 1 [file biology-11-01200-s001.zip › Supplementary Table S6.pdf]

Table S6. Percent (%) identity and similarity of amino acid sequences of three GnRH isoforms of small yellow croaker with those of other selected teleost species. Identity and similarity of corresponding GnRH isoform are shown with bold letters.

GnRH forms	Species	Percent (%) Identity & Similarity						NCBI Protein ID
		**Small Yellow Croaker**						
		**GnRH1**		**GnRH2**		**GnRH3**		
		Idnt.	Sim.	Idnt.	Sim.	Idnt.	Sim.	
GnRH1 (sb)	Large yellow croaker	**97.78**	**98.89**	24.47	41.49	17.35	35.71	NP_001290285
GnRH1 (sb)	Atlantic croaker	**96.67**	**98.89**	24.47	41.49	17.35	35.71	AAQ16501
GnRH1 (sb)	Japanese mackerel	**60.61**	**71.72**	28.00	40.00	17.65	30.39	ADP89591
GnRH1 (md)	Japanese medaka	**43.16**	**56.84**	20.00	31.58	15.31	32.65	BAB16303
GnRH1 (m)	Japanese eel	**37.98**	**54.74**	26.88	44.09	19.59	34.02	BAA82608
GnRH1 (wf)	Laker whitefish	**35.79**	**51.58**	25.53	43.62	22.45	35.71	AAP57221
GnRH1 (cf)	African catfish	**25.00**	**39.13**	25.84	42.70	18.75	32.29	CAA54971
GnRH1 (hr)	Japanese anchovy	**22.58**	**41.94**	26.67	47.78	22.45	34.69	AFO70217
GnRH2 (cGnRH-II)	Large yellow croaker	24.47	42.55	**100.00**	**100.00**	25.00	42.71	AIU45792
	Atlantic croaker	23.40	42.55	**98.82**	**100.00**	25.00	42.71	AAQ16502
	Japanese mackerel	24.47	41.49	**94.12**	**95.29**	27.08	42.71	ADP89592
	Japanese medaka	23.66	40.86	**78.82**	**83.53**	24.21	37.89	BAB16300
	Japanese eel	25.53	45.74	**68.97**	**82.76**	25.00	43.75	BAA82609
	Laker whitefish	23.40	43.62	**81.40**	**86.05**	27.08	40.63	AAP57219
	African catfish	20.21	38.30	**68.60**	**81.40**	27.08	39.58	CAA54969
	Japanese anchovy	23.40	42.55	**67.44**	**84.21**	26.04	43.75	AFO70218
GnRH3 (sGnRH)	Large yellow croaker	18.18	37.37	24.24	40.40	**97.78**	**98.89**	AJY54663
	Atlantic croaker	19.19	37.37	24.24	40.40	**95.56**	**97.78**	AAQ16503
	Japanese mackerel	17.17	35.35	21.21	38.38	**90.00**	**92.22**	ADP89593
	Japanese medaka	20.20	38.38	22.22	42.42	**82.22**	**90.00**	BAB16302
	Laker whitefish	15.79	31.58	18.48	36.96	**63.33**	**72.22**	AAP57220
	Japanese anchovy	23.53	37.25	17.65	33.33	**37.11**	**47.42**	AFO70219

*Idnt., Identity; Sim., Similarity
